# Cloud Based Fault Diagnosis by Convolutional Neural Network as Time–Frequency RGB Image Recognition of Industrial Machine Vibration with Internet of Things Connectivity

**DOI:** 10.3390/s23073755

**Published:** 2023-04-05

**Authors:** Dominik Łuczak, Stefan Brock, Krzysztof Siembab

**Affiliations:** Faculty of Automatic Control, Robotics and Electrical Engineering, Poznan University of Technology, 60-965 Poznań, Poland

**Keywords:** image recognition, HTTP, MQTT, feature extraction, convolutional neural network, time–frequency domain, short-time Fourier transform, sliding discrete Fourier transform

## Abstract

The human-centric and resilient European industry called Industry 5.0 requires a long lifetime of machines to reduce electronic waste. The appropriate way to handle this problem is to apply a diagnostic system capable of remotely detecting, isolating, and identifying faults. The authors present usage of HTTP/1.1 protocol for batch processing as a fault diagnosis server. Data are sent by microcontroller HTTP client in JSON format to the diagnosis server. Moreover, the MQTT protocol was used for stream (micro batch) processing from microcontroller client to two fault diagnosis clients. The first fault diagnosis MQTT client uses only frequency data for evaluation. The authors’ enhancement to standard fast Fourier transform (FFT) was their usage of sliding discrete Fourier transform (rSDFT, mSDFT, gSDFT, and oSDFT) which allows recursively updating the spectrum based on a new sample in the time domain and previous results in the frequency domain. This approach allows to reduce the computational cost. The second approach of the MQTT client for fault diagnosis uses short-time Fourier transform (STFT) to transform IMU 6 DOF sensor data into six spectrograms that are combined into an RGB image. All three-axis accelerometer and three-axis gyroscope data are used to obtain a time-frequency RGB image. The diagnosis of the machine is performed by a trained convolutional neural network suitable for RGB image recognition. Prediction result is returned as a JSON object with predicted state and probability of each state. For HTTP, the fault diagnosis result is sent in response, and for MQTT, it is send to prediction topic. Both protocols and both proposed approaches are suitable for fault diagnosis based on the mechanical vibration of the rotary machine and were tested in demonstration.

## 1. Introduction

Modern factories, cites, and households are equipped with an increasing number of electromechanical systems, which consume energy and have limited lifetimes. The appropriate maintenance of these devices can increase their useful life, which is cost-effective and environmentally friendly, reducing the number of electronic waste. In the literature, the term “electronic trash” or “trash” can also be found [1,2,3], but more often it is “electronic waste”, “e-waste” [4,5] or WEEE (Waste Electrical and Electronic Equipment) [6,7,8]. Maintenance of industrial machines is more demanding due to the complexity of electrical and mechanical parts. Perspective maintenance allows to avoid the unwanted stoppage of the production cycle and prevents unwanted damages of equipment. Deep understanding of available tools and the application of fault diagnosis is more demanding with the number of scientific articles already published in the field. Under keyword “fault diagnosis”, Google Scholar lists nearly 1.6 million articles. Limitation of results to “industrial machines” with operator AND gives 1.6 thousand articles. Another aspect of modern factories, cities, and households is increasing capability of exchange data through the global Internet network, which is named IoT (Internet of Things) or more narrowly, IIoT (Industrial Internet of Things) for industrial interconnections. Another term is Industry 4.0 which “describes the organization of production processes based on technology and devices autonomously communicating with each other along the value chain” [9,10]. However, the new term “Industry 5.0” was introduced towards a sustainable, human-centric and resilient European industry [8]. The publication database with the keyword “fault diagnosis” and “IoT” gives almost 12.3 thousand publications in the field. Therefore, both areas of fault diagnosis and IoT are popular and a growing field in the research area. The review of the patent database gives a new view on IoT and fault diagnosis. Since January 2020, the IPC (International Patent Classification) has been extended with the subclass G16Y titled “information and communication technology specially adapted for the internet of things”. Additionally, subgroup G16Y40/00 “IoT characterized by the purpose of the information processing” is dedicated to maintenance and management. Further detailed classification symbols are G16Y40/10 for detection; monitoring, G16Y40/20 analytics; diagnosis and G16Y40/40 maintenance of things. The Espacenet patent search gives nearly 2.8 thousand patents in a search by classification symbols G16Y40/10, G16Y40/20, and G16Y40/40. In the following sections, the author makes efforts to present a brief review of fault diagnosis and IoT, however, due to the large number of articles and patents, the limited pages of articles, and the limited time resources of the author, some aspects were omitted.

The manuscript is organized as follows: In chapter 2, general structure of the data collection system with local and Internet connectivity is described, which gives a view of the communication aspects of the fault diagnosis system with IoT for batch and stream data processing; in chapter 3, in detail are described IoT protocols such as HTTP (Hypertext Transfer Protocol) and MQTT (Message Queuing Telemetry Transport) for batch and stream data processing and their usage for data ingestion and data routing in the fault diagnosis system; in chapter 4, the structure of the fault diagnosis system from the signal processing point of view is presented; in chapter 5, different feature extraction methods that can be used for diagnosis in frequency domain such as FFT (fast Fourier transform) algorithms (radix-2 and radix-4), STFT (short-time Fourier transform), and group of algorithms of SDFT (sliding discrete Fourier transform) are explained; in chapter 6, a demonstration rig with MQTT connectivity is presented with dataset description in the time domain, frequency domain, and time–frequency domain. Based on preliminary selected single-axis data (gyroscope *Z* axis) in frequency domain features calculated by SDFT, a classifier was trained and tested which is an enhancement and reduction of computation requirements in comparison to features calculated by STFT; in Chapter 7, a proposed concept of usage 3-axis acceleration and 3-axis gyroscope sensor to create RGB (red, green, and blue) images from six time–frequency domain features which can be recognized by CNN (convolutional neural network) is presented and verified. The proposed approach proves that CNN can be successfully used in multispectrogram recognition which are organized into RGB images for fault diagnosis without the need for preliminary selection of the vibration axis. The proposed recognition of the RGB six-spectrogram and enhanced usage of SDFT instead of STFT was compared with other available methods and the results are shown in Table 1. CNN is used in vision-based recognition and vision applications [11,12,13,14]. Therefore, CNN can be applied to recognize specially prepared time–frequency images, as shown in the proposed method.

## 2. General Structures of Data Collection and Processing for Fault Diagnosis

**Figure 1:**
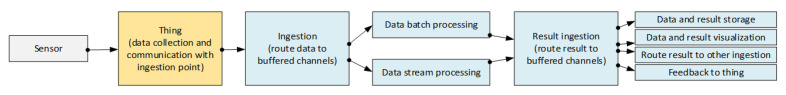
General structure of data pipelines in fault diagnosis.

The IoT maintenance system is based on measurements delivered in time series to the cloud. The most important part is to prepare data pipelines for fast transmission from sensors to the fault diagnosis server. At low level, analogue sensors are handled by ADC (analogue-to-digital converter). However, modern sensors are equipped with ADC and communication interfaces. Therefore, the sensor is connected to thing (microcontroller or microcomputer) by communication interface like SPI (Serial Peripheral Interface), I^2^C (Inter-Integrated Circuit), UART (universal asynchronous receiver-transmitter) or another device-specific communication interface. In the first stage of buffer, the classical producer–consumer application is applied between the sensor and the thing (microcontroller) where the sensors are equipped with internal buffers of several samples. The sensor is a producer of data and the thing is a consumer. The next stage is the use of data by microcontroller internal algorithms. The next stage of buffering is a producer–consumer application where the microcontroller is a data producer and the consumer is a cloud (server)-based fault diagnosis system. In each stage of data processing, the producer–consumer uses buffer for data. The general structure of the buffer-connected data pipelines is presented in Figure 1. Modern buffers offer routing to selected destinations which lead to processing in chain by micro services which are easy to modify compared to change in one large service. The collected data can be processed by the fault diagnosis system in two general ways, (a) batch processing or (b) stream processing. Batch processing is performed with data collected in a time window, but not often. Data stream processing is similar to batch processing but is performed repetitively in micro batch and can use the result of the previous micro batch result in the current micro batch processing.

**Figure 2:**
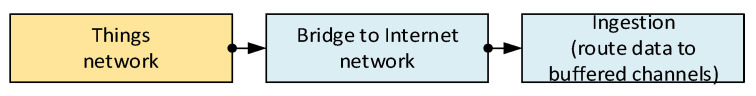
General network structure with network bridge.

**Figure 3:**
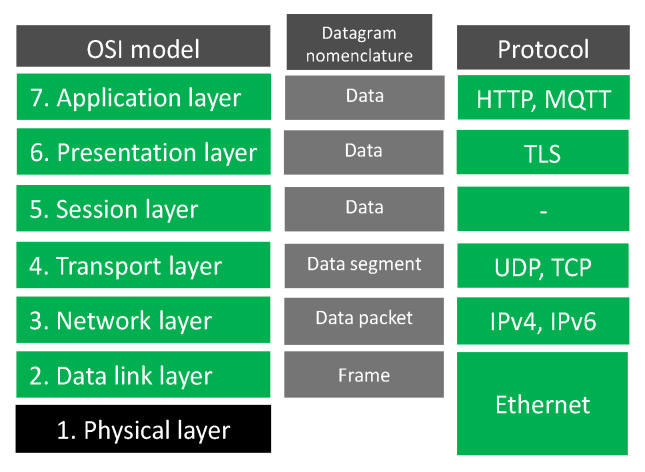
Model OSI-ISO Open System Interconnection—Open Source Initiative.

The general structure of data pipelines presented does not explain which protocol is used by the thing to ingest data to the cloud. Data collected in the thing network called OT (Operational Technology) are ingested into the cloud in the IT (Information Technology) network by the bridge system (Figure 2). OT part of the network is very wide. In IIoT, fieldbus interfaces can be CAN (Controller Area Network), RS232 (Recommended Standard 232), RS485 (Recommended Standard 485), industrial Ethernet, HART (Highway Addressable Remote Transducer), and other physical layers. The main fieldbus technologies used in industry are presented in the IEC (International Electrotechnical Commission) 61784-1:2019 standard [19]. The 2019 standard groups communication technologies into 19 CPF (Communication Profile Families). The CPFs are divided into 24 profiles: Foundation™ Fieldbus H1 (31.25 kbit/s), HSE (High-speed Ethernet); ControlNet, Ethernet/IP™, DeviceNet^®^, Profibus DP (Decentralized Peripherals) and PA (Process Automation), P-NET^®^, WorldFIP^®^ which has 3 profiles, Interbus^®^ which has 3 profiles, CC-Link (Control and Communication Link) which has 3 profiles, HART^®^, WirelessHART^®^, SERCOS (acronym for SErial Realtime COmmunications System) I and II, and Mechatrolink-II and M-III. Each CPF profile has described the physical layer, the data link layer, and the application layer of the OSI-ISO (Open System Interconnection—Open Source Initiative) model (Figure 3). The IT network has adopted well the network and transport layer by IPv4 (Internet Protocol version 4) [20], IPv6 (Internet Protocol version 6) [21] and connectionless UDP (User Datagram Protocol) [22], and connection-oriented TCP (Transmission Control Protocol) [23], respectively. Layers above the transport layer are described in the next section. The link layer used by physical and data is Ethernet defined by standard 802.3-2022 [24] approved on 13 May 2022. The physical layer allows for the use of operation over coaxial, twisted pair, or fiber optic cables, or electrical backplanes. The Ethernet standard covers speeds of operation from 1 Mb/s to 400 Gb/s.

## 3. Internet of Things Protocols for Batch and Stream Processing of Fault Diagnosis

**Figure 4:**
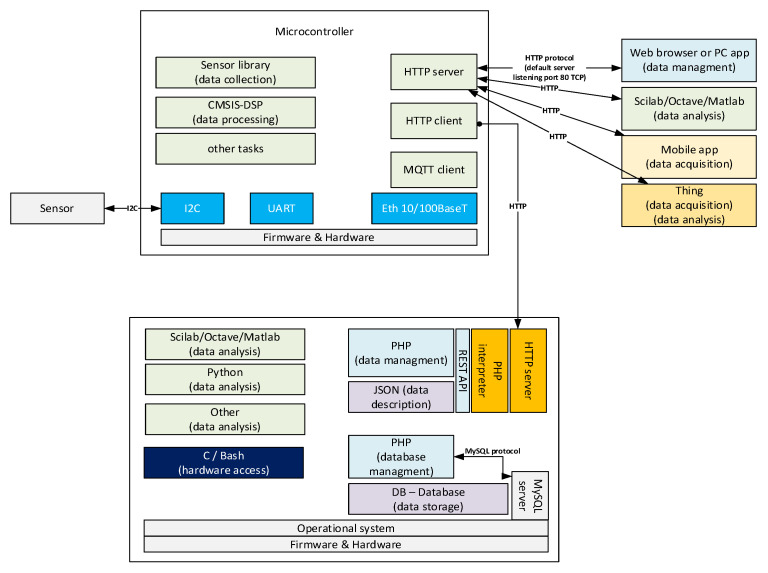
Client–server architecture with HTTP/1.1.

The protocols used in the presentation layer in OSI-ISO model (Figure 3) are generally responsible for ensuring the security of transmission in three main aspects: authentication, confidentiality, and integrity of data. The fault diagnosis server side of the channel is always authenticated, and the client (microcontroller) side is optionally authenticated. Confidentiality means that the data sent over the channel after establishment is only visible to the endpoints. Integrity means that data sent over the channel after establishment cannot be modified by attackers without detection. The latest version of TLS (Transport Layer Security) is 1.3 [25] presented in 2018. The layer allows us to use asymmetric cryptography or symmetric cryptography with a pre-shared key. The highest level in the reference model is the application layer, where the HTTP (Hypertext Transfer Protocol) and MQTT (Message Queuing Telemetry Transport) protocols are widely used in IoT applications. The text-based HTTP protocol was widely used in version 1.1 [26] developed in 1997 [27]. HTTP/1.1 can be upgraded to the WebSocket protocol [28], which is useful for established connections to exchange data streams in a bidirectional manner. The next version 2.0 of HTTP was shown in 2015 [29], where the protocol was changed to binary encoding instead of text. The newest version 3.0 of HTTP was introduced in June 2022 [30]. HTTP/3 uses a new transport layer protocol QUIC (Quick UDP Internet Connections) [31] standardized in 2021 instead of TCP. The QUIC transport protocol uses UDP and TLS to provide a stream of binary frames between client and server. Streams can be intended by the client or server as unidirectional or bidirectional. The HTTP/1.1 protocol can be adopted in the batch processing of faults as the request–response principle (Figure 1). The connection is closed by the fault diagnosis server after the response has been sent to the client request. HTTP/1.1 upgraded to WebSocket, HTTP/2, and HTTP/3 are suitable for the stream processing principle (Figure 1) because the connection is not closed after the server sends the response. The HTTP server can be implemented on the microcontroller (thing) or on the remote computer (cloud), see Figure 4.

**Figure 5:**
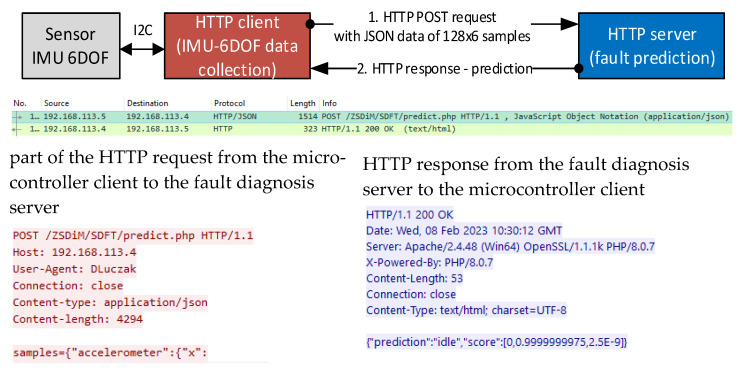
HTTP/1.1 request–response as batch processing of machine state prediction.

Fault diagnosis as batch processing with HTTP/1.1 request–response header is shown in Figure 5. The microcontroller acts as an HTTP client which sends sensor-collected data in JSON (JavaScript Object Notation) format of 4294 character length by the POST method. The server path is */*…*/predict.php* in the request line. The HTTP server responds *200 OK* in the status line, which means that the data were processed correctly and machine state prediction is given in the response message body in JSON format. Presented response contains predicted class “*prediction*”*:*“*idle*” and probability “*score*” of each class as array in order: *normal, idle*, and *fault*. 

**Figure 6:**
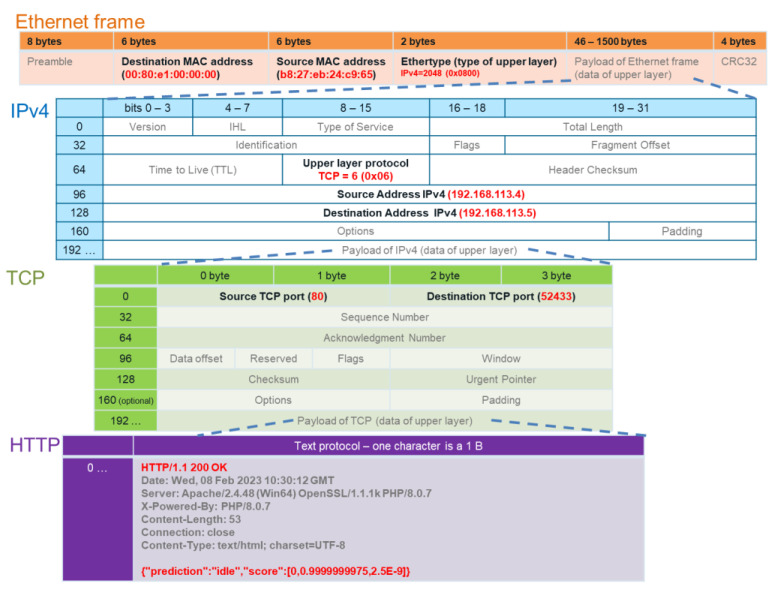
Response from the fault prediction server with HTTP/1.1 encapsulated in the lower layers of the OSI-ISO model.

Data from the application layer are encapsulated by lower layer headers. Figure 6 presents the encapsulation of HTTP/1.1 response from server to client. Data from HTTP/1.1 layer are put into the field payload of TCP and the header of TCP needs to be filled. The default TCP port is 80 for the HTTP/1.1 server.

The data segment is then a payload for IPv4, and the IPv4 header needs to be filled. The IPv4 header field upper-layer protocol is set to 6. The destination address and the source address must be known. The last encapsulation is into an Ethernet frame where the data packet is a payload of the Ethernet frame, and the header of the Ethernet needs to field. The hardware MAC (media access control address) destination address and source address need to be known. If only the IPv4 destination address is known before sending the Ethernet frame, the MAC destination address must be resolved by the ARP (Address Resolution Protocol) [32].

**Figure 7:**
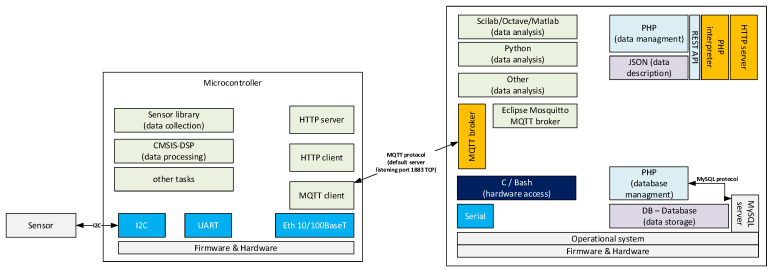
MQTT 3.1 client–server architecture.

The other application layer protocol adopted for IoT applications is MQTT in version 3.1.1 [33] published in 2015 and version 5.0 [34] updated in 2019. MQTT provides client–server publish/subscribe messaging (Figure 7). The client connects to the server (broker) and subscribes to the topic. Messages published by other clients to the same topic will be pushed to all clients subscribed to this topic by the broker (server). MQTT use TCP at transport layer which establishes a connection as long as the client requires. The default destination TCP port of the broker is 1883.

**Figure 8:**
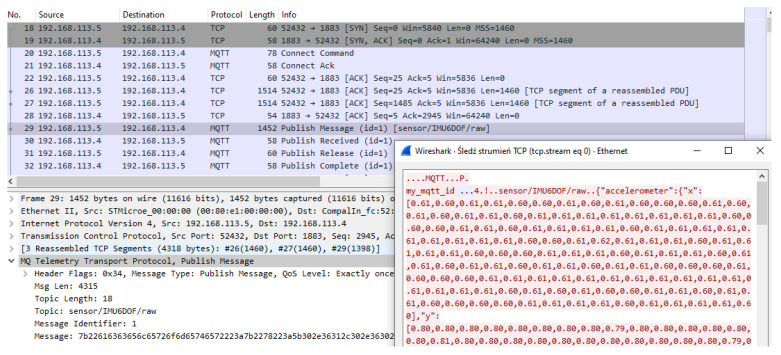
Captured MQTT v3.1 packets captured with TCP handshake for microcontroller MQTT client write sensor data to the topic *sensor/IMU6DOF/raw* as stream processing (micro batch) for machine state prediction.

The three step TCP handshake from the 52432 client port to the 1883 server port is presented in Figure 8 to establish the connection. After successful subscription, the MQTT microcontroller client send messages to other clients through the broker. The topic can be treated as a buffer name in the producer–consumer architecture. The designed fault diagnosis system consists of two MQTT clients subscribed to topics: *sensor/IMU6DOF/raw* and *sensor/IMU6DOF/predict*. The microcontroller MQTT client writes sensor data to the topic *sensor/IMU6DOF/raw*, and the MATLAB MQTT client retrieves those samples in JSON format. Received samples are transformed into features by digital signal processing methods, which are then evaluated by the classification algorithm. The prediction of classification algorithm is transformed into JSON (e.g., {“prediction”:“idle”,“score”:[0,0.9999999955,4.5E-9]}) and sent to *sensor/IMU6DOF/predict* topic.

MQTT can be used in either stream and batch data processing (Figure 1) of a fault diagnosis system. It is important to note that all messages are sent throughout the broker, which leads to central-oriented messaging with routing possibilities. Many clients who subscribed to the same topic received the same data. Therefore, clients can perform different micro services on the same data stream like feature extraction, data storage in a database, or visualization of data stream. This leads to transmission of one-to-many (one-point to multiple points) messages. In contrast to MQTT, WebSocket allows only point-to-point data streaming without the built-in message routing mechanism.

Another protocol used in IoT applications is CoAP (Constrained Application Protocol), defined in 2014 [35]. The protocol is dedicated to 8-bit microcontrollers with limited RAM (random access memory) and ROM (read-only memory). CoAP typical applications include smart energy and building automation. The direct quote from study [35] follows: “The goal of CoAP is not to blindly compress, but rather to realize a subset of REST (representational state transfer) common with HTTP but optimized for M2M (machine-to-machine) applications.” Many similarities to HTTP can be found. Similarly to HTTP, the CoAP uses request/response model with GET, PUT, POST, and DELETE methods. In contrast to HTTP, the CoAP uses the UDP protocol in the transport layer (see Figure 3). The use of UDP protocol in transport layer is unreliable which means that it does not handle missing packets, duplicate packets or packets retrieved in a different order caused by a different path of packets in the Internet. Therefore, in 2018 [36], CoAP was designed to use TCP, TLS, and WebSocket transports.

According to the *Content-Type* (see Section 3.1.1.5 in [26]) and *Content-Encoding* (see Section 3.1.2.2 in [26]) part in HTTP 1.1, many different data types are allowed in the message body part. Commonly adopted content types for the IoT application are JSON (JavaScript Object Notation) [37,38], XML (Extensible Markup Language) [39] or raw bytes. The full list of the allowed media types [40] is published by IANA (Internet Assigned Numbers Authority) in [41]. More than 1500 available formats are in the single application section of *Media Types* published on 21 November 2022. All allowed formats are grouped into sections, which are as follows: application, audio, font, example, image, message, model, multipart, text, video. Up-to-date XML media types “application/xml” or “text/xml” were published in 2014 [42] and the up-to-date JSON media type “application/json” was published in 2017 [43].

## 4. General Structure of Fault Diagnosis and Perspective Maintenance

**Figure 9:**
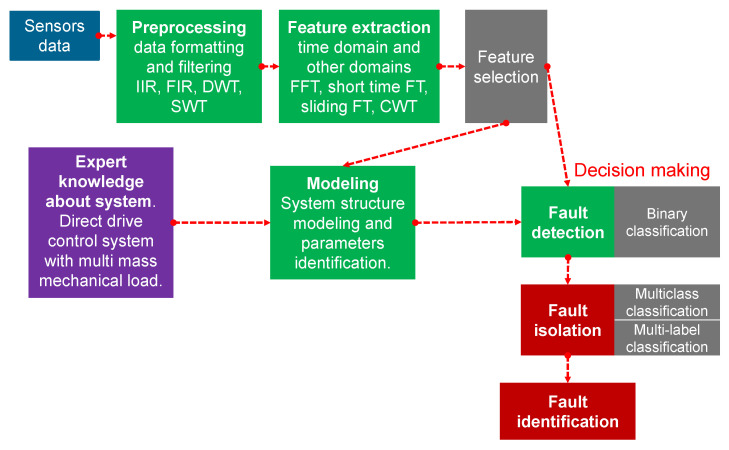
General structure of the fault diagnosis system.

The fault diagnosis system (Figure 9) detects faults based on changes in features over time. The fault diagnosis system works with the client–server architecture in the IT network [44,45,46,47,48,49,50]. The features are extracted from raw measurements and data or pre-processed data. Fault detection means recognition in the change of machine state caused by one or more faults. It can be treated as anomaly state detection, so any state different from normal behavior can be detected. Fault isolation is the recognition of which parts of the machine have faults. Finally, fault identification answers how wide the damages are. The information about the fault can be used by the maintenance team to repair the system. However, if repair is not possible immediately and the system cannot be stopped safely, then FTC (fault tolerant control) is a key component. The system can have hardware redundancy, which will be used if failure occurs, however, it is expensive to duplicate parts and not always technically possible to apply. FTC allows to continue the system usage of the system with performance constrains [51,52,53,54,55,56].

Fault diagnosis can be made only on data, which is called a data-driven approach. Another option is to prepare a mathematical model of the diagnosed system and analyze the residual data between the model and the system. Today, the investigated system model is called a digital twin [57,58,59,60,61,62,63,64]. Modeling is a very wide area in which static behavior [65,66,67], dynamic changes and continuous system [68,69], and discrete states [70] of systems can be modeled. Contrary to the analysis of model and real system residuum is the analysis of estimated parameters of system. The fault diagnosis system compares the estimated parameters in a predefined bounded region. The fault is announced if one of the parameters is outside the defined boundaries longer than the chosen amount of time.

The sensor presented in Figure 9 can be any kind. It can be an additional sensor only for fault diagnosis or a sensor already present in the system used by the control algorithms. The electromechanical machine or power system can be investigated by many different sensors and signals: current [71,72] and voltage [73,74], torque [75,76], angular velocity/position [77,78], linear 3-axis acceleration/speed/position [16,17], Doppler laser vibrometer [79], transmission coefficient and reflexion coefficient of omnidirectional antenna [80], strain/tension [81,82,83,84], power consumption [85,86,87,88], internal/external temperature at selected points [89,90] or surface temperature by thermal camera [91,92], depending on frequency range: displacement [93], vibrations [15,18,94,95,96], sound [97,98,99], sound from several microphones [100] or ultrasound [101,102], vibro-acoustic [103], chemical analysis of lubrication [104,105], chemical analysis by spectral imaging [106,107,108,109], camera imaging in human colour spectrum [110,111,112,113], and signals to virtual image [114,115,116,117,118].

## 5. Feature Extraction Methods

The decision made by the fault diagnosis system can be taken based on data in the time domain [119,120] or other domains, for example, the frequency domain [121,122,123,124], the time–frequency domain [125,126,127,128,129,130,131] or the time-scale domain [132,133,134,135]. 

Analysis with frequency domain is achieved by using one of the FFT (fast Fourier transform) algorithms. These algorithms decompose the analyzed signal into sinusoidal/cosinusoidal components at frequency from 0 Hz up to half of the sampling frequency. The use of the fast Fourier transform algorithm radix-2 [136,137] requires a number of samples equal $N=2^{k},k\in N$. However, the radix-4 algorithm requires $N=4^{k}$ [138,139]. The authors in their publications mostly inform the reader about the use of FFT without mentioning the algorithm used [140,141,142]. Frequency analysis can be applied to stationary signals in one time window. On the other hand, if knowledge about the sinusoidal/cosinusoidal components and time of their duration in the analyzed signal is required, then time–frequency analysis needs to be applied.

The use of FFT on short time-shifted windows leads to the STFT (short-time Fourier transform) method. STFT changes a one-dimensional time series signal into a two-dimensional time and frequency [143,144,145]. The STFT parameters are as follows: time window shape (e.g., rectangle, Hamming [146,147], Hanning [148,149]), step size or overlap of next time window that influence time resolution, time window length that influence frequency resolution $f_{res}=f_{s}/N$, where N—signal length in samples and $f_{s}$—sampling frequency. Selection of time window length is a trade-off between good time localization or good frequency localization of the sinusoidal/cosinusoidal components. STFT requires an FFT calculation on each shifted time window. If the step size is equal to 1, it means that FFT will be calculated on N−1 old and 1 new samples, which is not computationally effective. The problem of spectrum update at each new sample was solved using the SDFT method (sliding discrete Fourier transform).

SDFT was presented in 1997 [150] and well described in 2003 (rSDFT, 2003) [151] with an update in 2004 (rSDFT, 2004) [152]. Using study [152], the single spectrum component $S_{k}$ at frequency k is calculated by $S_{k}\left(n\right)=e^{j2\pi k/N}(S_{k}\left(n-1\right)+x\left(n\right)-x\left(n-N\right))$, where N—signal length in samples, n—time index, x—signal value. The discrete transfer function of study [152] is $H_{rSDFT,2004}\left(z\right)=e^{j2\pi k/N}\left(1-z^{-N}\right)/(1-e^{j2\pi k/N}z^{-1})$, which allows for stability analysis of the algorithm. In 2010, mSDFT (modulated SDFT) was introduced [153]. In study [153], SDFT 2003 with the 2004 update was named rSDFT (recursive SDFT). The mSDFT compared to rSDFT-2004 has reduced the accumulated error and potential instabilities. The stability of algorithms was analyzed and a new guaranteed stable SDFT algorithm (gSDFT) was published in 2015 [154]. The gSDFT algorithm has slightly smaller accumulated errors and better performance compared to mSDFT. Other improvements of mSDFT, called cascade integrator comb (CIC)—SDFT, which allow the embedded usage of the B-spline window function, were presented in 2017 [155]. In the same year, optimal sliding DFT (oSDFT) was presented [156], which reduces the number of multiplications by 73.44%, 64.58%, 82.30%, and 29.17% as compared to FFT, rSDFT, mSDFT, and gSDFT, respectively. The oSDFT accelerates the sliding transform process by 57.02%, 7.51%, 39.77%, and 9.38% compared to the FFT, rSDFT, mSDFT, and gSDFT algorithms, respectively. Misleading may be the name oSDFT (observer-based SDFT) introduced in 2018 [157], which uses the state observer method for digital signal processing purposes.

## 6. Demonstration of Fault Diagnosis with MQTT Communication

**Figure 10:**
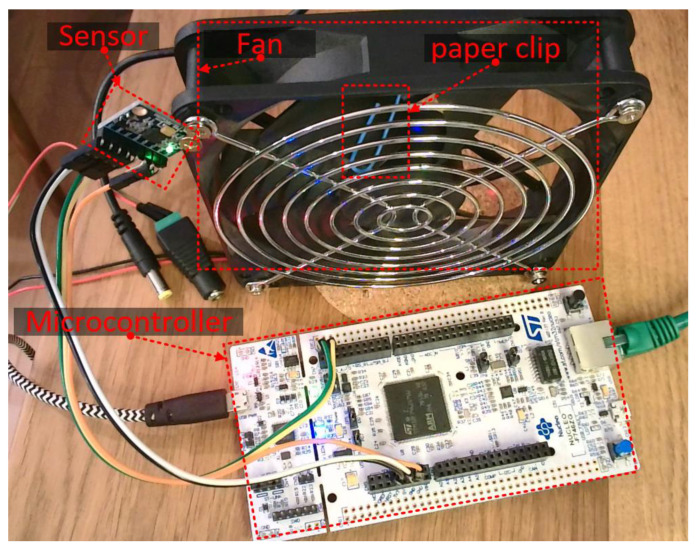
Demonstration system for fan fault diagnosis.

The demonstration shows classification of computer fan work into classes: *idle*, *normal*, or *fault*. The failure is caused by a paper clip added to the fan blade. The prepared demonstration setup (shown in Figure 10) consists of a NUCLEO board with STM32F746ZG microcontroller that handles the IMU-6 DOF (inertial measurement unit with 6 degrees of freedom) MPU6050 sensor. Data are collected synchronously with constant sampling time equal to 5 ms (sampling frequency 200 Hz). The vector of 128 samples from the 3-axis accelerometer and 3-axis gyroscope is then transformed into JSON (JavaScript Object Notation) as the object {“accelerometer”:{“x”:[],“y”:[],“z”:[]},”gyroscope”:{“x”:[],“y”:[],“z”:[]}}, where the array “[]” contain samples. Data are sent on the topic “sensor/IMU6DOF/raw” by the MQTT client implemented in the microcontroller to the MQTT broker hosted on a personal computer. Eclipse Mosquitto™ was used as an MQTT broker [158]. The data stream was processed in real time by MATLAB R2022b with the MQTT client [159]. 

**Figure 11:**
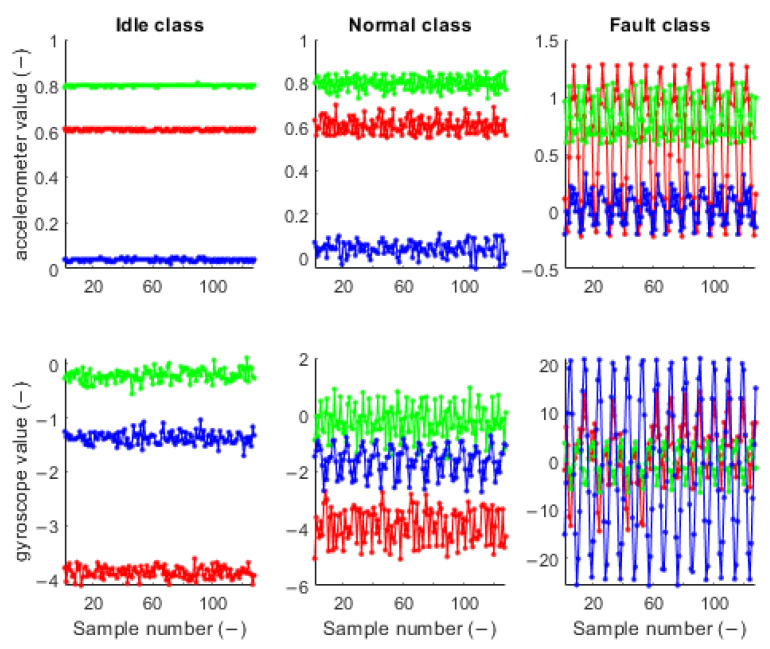
Accelerometer and gyroscope time domain data in 3-axis: red—*X*, green—*Y*, and blue—*Z*.

**Figure 12:**
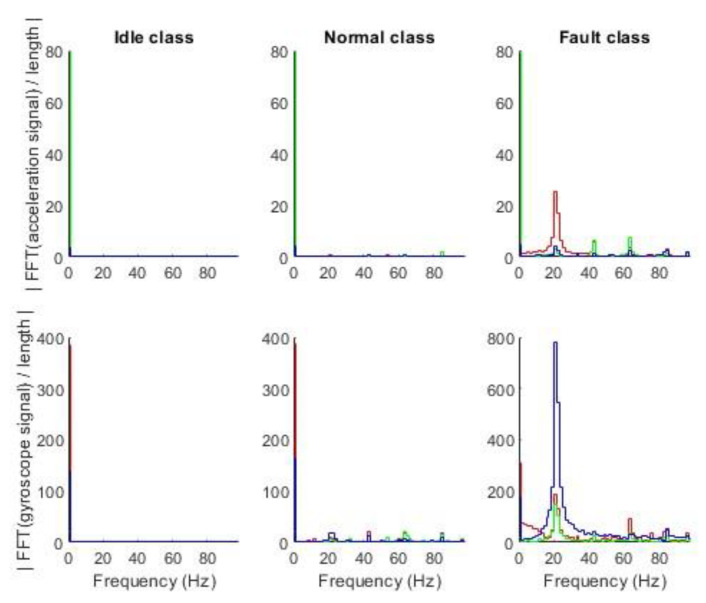
Accelerometer and gyroscope frequency domain data in 3-axis: red—*X*, green—*Y*, and blue—*Z*.

The first stage is data collection at predefined class states for several seconds. Data collected in one micro batch in time domain are shown in Figure 11**.** Data were collected for three states of the fan: (1) *idle* class—fan power is switched off, (2) *normal* class—fan power without paper clip is switched on, and (3) *fault* class—fan power with paper clip is switched on. The time domain signal for the *idle* class has a constant signal with noise, the signal in the *normal* class has small oscillations, and the signal in the *fault* class has significant oscillations compared to previous classes. Data were transformed to the frequency domain by the FFT function in MATLAB, resulting in a complex number vector. The absolute value of the complex numbers is shown in Figure 12. Each class has different frequency features in the range of 0 Hz to 100 Hz. The most significant change in frequency occurs on the *Z* axis of the gyroscope.

**Figure 13:**
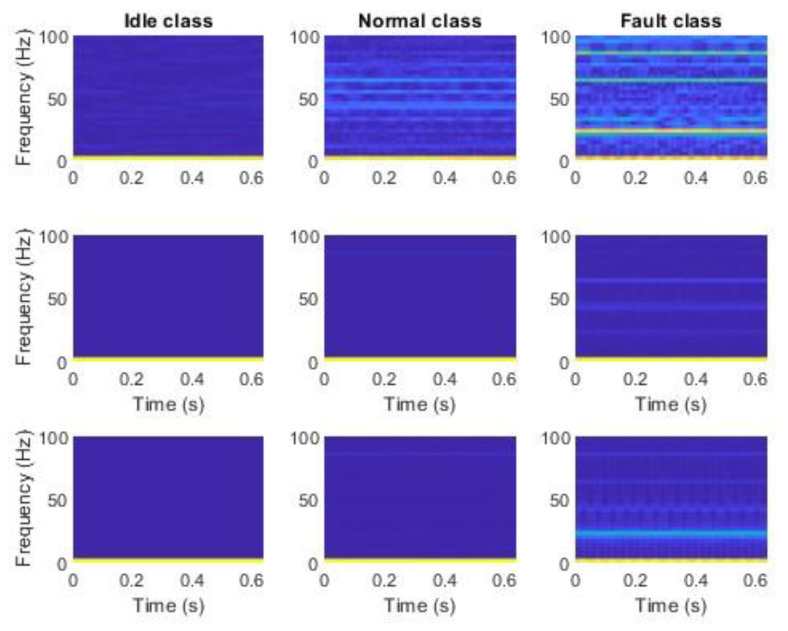
Accelerometer data in the time–frequency domain using mSDFT in the 3-axis: **top** row, *Z*, **middle** row, *Y*, **bottom** row—*X*.

**Figure 14:**
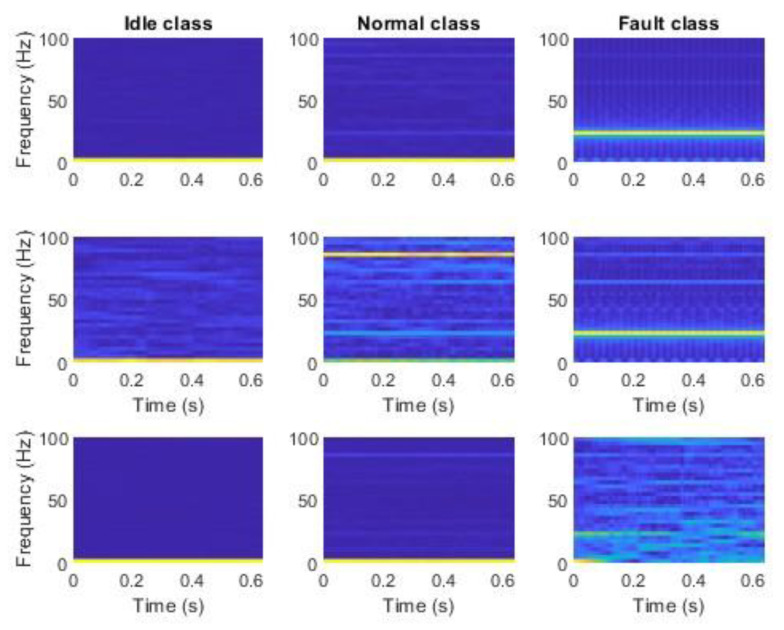
Gyroscope data in the time–frequency domain using mSDFT in 3-axis: **top** row—*Z*, **middle** row—*Y*, **bottom** row—*X*.

The second stage of preparation was the extraction of features using mSDFT [153]. Each step of mSDFT returns 33 complex numbers at frequencies in the range from 0 Hz to 100 Hz. The algorithm was implemented in MATLAB and the absolute values of the complex number were stored in the files. Each row of the file contains 33 points that were used as a feature vector. The sliding discrete Fourier transform result can be gathered after each new sample. The multiple spectrums calculated at different times are shown in Figure 13 and Figure 14. The time–frequency analysis showed that for each class, the dominant frequency components are constant and do not vary with time.

**Figure 15:**
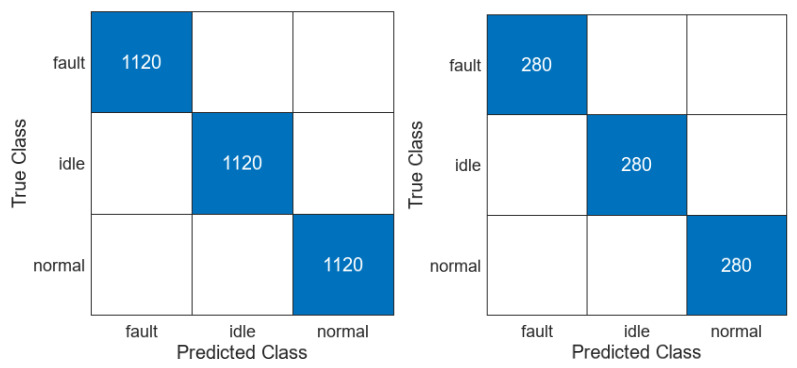
Confusion matrix of the trained classifier (train—**left**; test—**right**).

The third stage of the demonstration requires the preparation of a classification. The features saved in the files for each class were read and loaded into MATLAB Classification Learner [160]. Several classifiers were trained, and the best was used for real-time verification. The dataset has 4200 observation sets randomly divided into 80% training and 20% test dataset. The training process was carried out on 1120 sets of *fault* class, 1120 sets of *idle* class, and 1120 sets of *normal* class. One set consists of 33 frequency features calculated by mSDFT from the gyroscope Z axis signal. The confusion matrix of the trained classifier is shown in Figure 15. The classifier can be used by applying new calculated features to *predictFcn* in MATLAB. The prediction result is sent to the MQTT *sensor/IMU6DOF/predict* topic in the data pipeline (see Figure 1).

## 7. Recognition of a Time–Frequency RGB Image of Vibration

**Figure 16:**
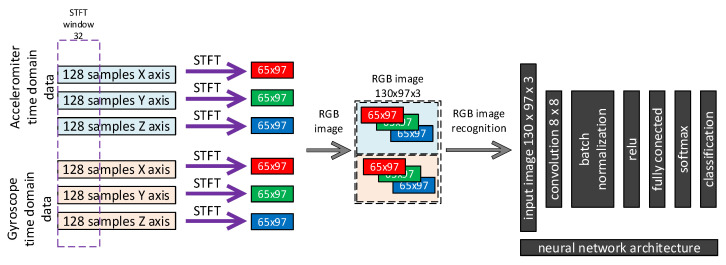
STFT RGB image creation and convolutional neural network architecture.

**Figure 17:**
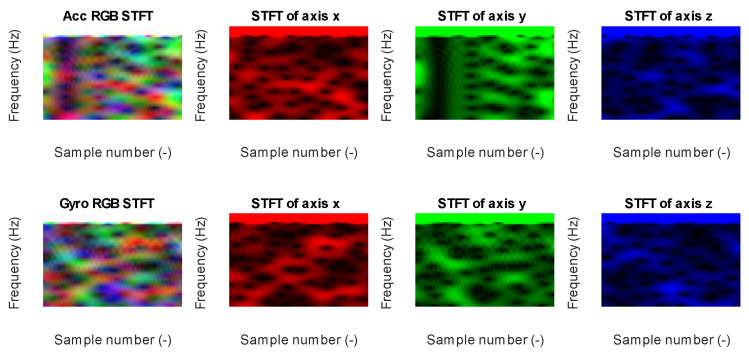
STFT RGB image of idle class for accelerometer *x*, *y*, and *z* axis (**top**) and gyroscope *x*, *y*, and *z* (**bottom**).

**Figure 18:**
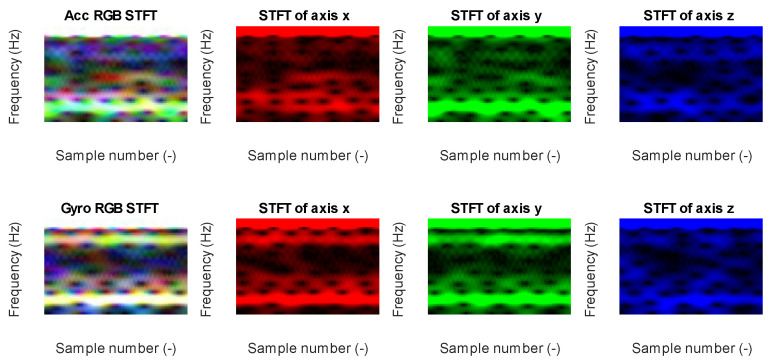
STFT RGB image of normal class for accelerometer *x*, *y*, and *z* axis (**top**) and gyroscope *x*, *y*, and *z* (**bottom**).

**Figure 19:**
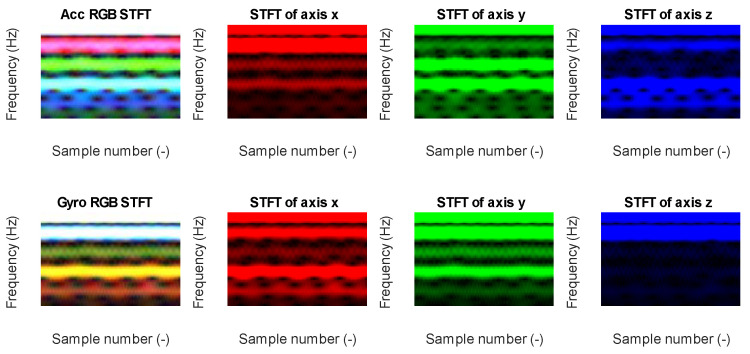
STFT RGB image of the fault class for the accelerometer *x*, *y*, and the *z* axis (**top**) and gyroscope *x*, *y*, and *z* (**bottom**).

**Figure 20:**
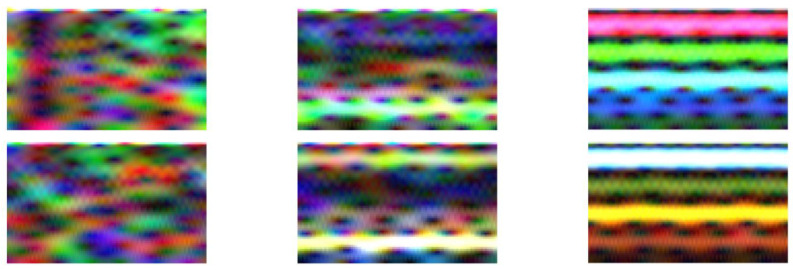
Time–frequency RGB images for class: idle (**left**), normal (**middle**), fault (**right**).

Data from each axis of IMU 6-DOF were transformed into the time–frequency domain by applying STFT (short-time Fourier transform) with 32 samples of window length and 31 samples of overlap (see Figure 16, Figure 17, Figure 18, Figure 19 and Figure 20). As a result of STFT at 128 time domain samples of single axis, there was a two-dimensional signal of 65 frequencies at 97 time moments. The process was repeated for each axis of accelerometer and gyroscope. Finally, six images for 128 × 6 samples of IMU 6-DOF were obtained in the time–frequency domain for each class: *idle* in Figure 17, *normal* in Figure 18, and *fault* in Figure 19. These six images are combined into one RGB (red, green, and blue) image of size 130 × 97 × 3 in Figure 16; representative RGB images for each class are shown in Figure 20.

**Figure 21:**
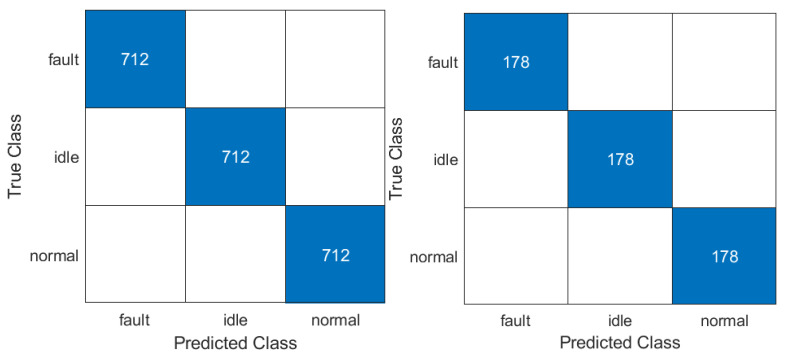
Confusion matrix of the trained (**left**) and tested (**right**) CNN classifier.

Data collected in the time domain for each class were converted into time–frequency RGB images. Together, the dataset has 2670 RGB images divided into classes: *fault* with 890 RGB images, *idle* with 890 RGB images, and *normal* with 890 RGB images. All images, which were divided into training and validation datasets. From the dataset, 80% is the training set (2136 RGB images) and 20% is the testing set (534 RGB images) which were randomly selected. CNN (convolutional neural network) training was performed in MATLAB Deep Learning Toolbox [161] with the support of NVIDIA GPU (graphics processing unit) with CUDA^®^ (Compute Unified Device Architecture). Validation of the trained convolution neural network confirms good classification, shown as a confusion matrix in Figure 21. 

## 8. Discussion

The cloud-based fault diagnosis system is suitable for human-centric and resilient European Industry 5.0 that requires a long lifetime of machines to reduce the amount of e-waste. A large number of industrial protocols used, of which only part are grouped in Communication Profile Families, underline the problem of data collection and ingestion into the cloud system. However, Internet protocols are developed separately without considering the needs of industrial processes. Therefore, the adoption of existing and new Internet protocols for industrial processes is a challenge. Batch and stream processing requires different handling. Batch processing can be applied with the request–response HTTP/1.1 protocol with a closing connection. However, stream processing requires an established connection for a long time, which can be achieved by WebSocket, HTTP/2.0, HTTP/3.0 or MQTT v3.1 and v5.0. It is worth considering the data exchange problem as a producer–consumer problem with a buffer in the middle. The producer can collect data from many different sensor technologies to investigate the normal and abnormal behavior of an industrial system. An interesting concept is the usage-only energy meter with the energy consumption profile for fault diagnosis. The decision about fault detection is made on the selected signal characteristics. A wide range of diagnostic applications, especially in rotating machines, use frequency analysis or time–frequency analysis. Therefore, short-time Fourier transform (STFT) and sliding discrete Fourier transform algorithms (rSDFT, mSDFT, gSDFT, and oSDFT) were underlined in the article. In batch processing, FFT (radix-2 or radix-4) or STFT can be applied. Real-time stream processing in the frequency domain can be performed using one of the SDFT algorithms.

The manuscript contains a demonstration of the fan fault diagnosis. Data were collected synchronously using a 3-axis accelerometer and a 3-axis gyroscope and microcontroller. The data collected were transformed into a JSON text structure. Those data in JSON are sent to an HTTP server for batch processing and as a result, fault diagnosis server returns a prediction in JSON format. Another investigation was conducted by sending data collection in JSON from the microcontroller client to the MQTT broker in real time as a stream (micro batch) on the *sensor/IMU6DOF/raw* topic for fault prediction. This approach allowed us to use two fault diagnosis predictors (two MQTT clients subscribed to *sensor/IMU6DOF/raw* topic) based on mSDFT and the second based on recognition of the time–frequency RGB image of vibration. For the first predictor, the time series data were transformed into the frequency domain by the mSDFT and evaluated by classification algorithm. The second predictor calculates six STFT and combines the result into an RGB image which is evaluated by the convolutional neural network. The result of both predictors is sent to *sensor/IMU6DOF/predict* topic in JSON format which contains the predicted state and probability for each class. This approach allowed us to classify the state of machine by two different algorithms and can be further extended by other algorithms to ensure voting of prediction from different algorithms to increase software algorithm redundancy.

## 9. Conclusions

The presented two communication methods allow for rapid prototyping of the fault diagnosis system with cloud connectivity. The HTTP server allows for one-to-one fault diagnosis, where data from one microcontroller are evaluated by one fault diagnosis server endpoint. On the other hand, MQTT allowed for one-to-many fault diagnoses when the same data were evaluated by two fault diagnosis clients with different feature extraction and different classification algorithms. HTTP and MQTT use TCP, however, due to connection closing in HTTP and connection remaining in MQTT, their work differently and both are suitable for fault diagnosis. In the authors’ opinion, it is more convenient to use MQTT for rapid prototyping and modularity of a variety of environments of the fault diagnosis system.

The authors have shown two approaches for fault diagnosis. One is based on frequency analysis which was enhanced in comparison to classical ones by usage of sliding Fourier transform (SDFT) instead of fast Fourier transform (FFT). This modification allows us to update the spectrum based on a new sample in time domain and spectrum in the previous step, which work as an IIR (Infinite Impulse Response) filter where filter input is one time domain sample and filter output is one frequency domain sample (complex number). The second proposed approach transforms data from IMU 6-DOF sensor into six spectrograms by STFT, which are used to create an RGB image. This time–frequency RGB image is evaluated by a convolutional neural network for fault diagnosis. The proposed approach proves that CNN can be successfully used in multispectrogram recognition which are organized into RGB images for fault diagnosis without the need for preliminary selection of a vibration axis.

Future research will be focused on increasing the TRL (technology readiness level). Research was carried out at TRL 1 to validate the proof of principles and concept. Further work will be conducted to increase the TRL to higher levels to validate in the laboratory environment the rotary electric machine (electric drive) with fault diagnosis system. At the next TRL, more faults of electric drive will be considered with different natures (electrical and mechanical), e.g., fault in inverter topology, fault in bearing, and shaft cracks.

## Figures and Tables

**Table 1:** Comparison of proposed and enhanced methods.

IoT Connectivity	Types of Fault and Labels	Signal or Sensor	Features Extraction Method	Features	Classificator	Article
MQTT and HTTP	demonstration of fan blades’ imbalance (normal, fan off, fan with fault)	3-axis accelerometer and 3-axis gyroscope	SDFT or STFT at six axis	RGB image made of six time–frequency domain data	CNN	Proposed
MQTT and HTTP	demonstration of fan blades’ imbalance (normal, fan off, fan with fault)	preliminary selected one axis of 3-axis accelerometer and 3-axis gyroscope	SDFT at one axis enhanced to STFT	frequency domain data	classical classifier	Enhanced
Not specified	bearing (normal, inner ring, outer ring, ball)	vibration one-axis	STFT	color spectrogram of one signal	CNN	[15]
Not specified	bearing normal and four faulty states (ball, inner ring, outer ring, inner + outer)	3-axis accelerometer	frequency transform with weight map	frequency domain for each axis	CNN	[16]
Not specified	blades non-damaged and two fault (5% and 15% broken blades)	from one axis to 3-axis of angular velocity	WPT (wavelet packet transform)—wavelet name not specified	third level of WPT decomposition	LSTM (long and short-term memory)	[17]
Not specified	bearing (normal, outer, ball, inner)	raw data is one-dimensional signal; sensor is not specified	CWT (continuous wavelet transform), STFT	CWT, time domain, and frequency domain features aggregation	MIMTNet (multiple—input, multiple—task CNN)	[18]

## Data Availability

Not applicable.
